# Slow Diffusion Underlies Alternation of Fast and Slow Growth Periods of Microtubule Assembly

**DOI:** 10.1155/2014/601898

**Published:** 2014-01-30

**Authors:** Ming Yang

**Affiliations:** Department of Botany, Oklahoma State University, 301 Physical Sciences, Stillwater, OK 74078, USA

## Abstract

*In vitro* microtubule assembly exhibits a rhythmic phenomenon, that is, fast growth periods alternating with slow growth periods. Mechanism underlying this phenomenon is unknown. Here a simple diffusion mechanism coupled with small diffusion coefficients is proposed to underlie this phenomenon. Calculations based on previously published results demonstrate that such a mechanism can explain the differences in the average duration of the interval encompassing a fast growth period and a slow growth period in *in vitro* microtubule assembly experiments in different conditions. Because no parameter unique to the microtubule assembly process is involved in the analysis, the proposed mechanism is expected to be generally applicable to heterogeneous chemical reactions. Also because biological systems are characterized by heterogeneous chemical reactions, the diffusion-based rhythmic characteristic of heterogeneous reactions is postulated to be a fundamental element in generating rhythmic behaviors in biological systems.

## 1. Introduction

A living system such as a cell consists of multiple heterogeneous catalytic reactions that occur at the solid-liquid interphase. Some of the most prominent examples of such reactions include microtubule assembly during spindle formation, protein synthesis on ribosomes, and DNA and RNA formation from existing DNA templates. It is generally accepted that diffusion rate can be a limiting factor for the product output of a heterogeneous reaction system [1, 2]. However, how diffusion affects a heterogeneous reaction involving a cellular structure has not been described with the support of experimental data.

The diffusion coefficient *D* of a molecule species, which defines the diffusion rate of the molecule in a solution, is determined by the size and shape of the molecule and the temperature and viscosity of the solution [3]. Diffusion coefficients of small and large molecules in cellular compartments have been experimentally determined, with the *D*s of proteins in the cytoplasm of various cells ranging from 0.15 to 40 *μ*m^2^/s [4–7]. The *D* of tubulin dimers in the cytoplasm of embryonic cells of sea urchin was found to be 4–10 *μ*m^2^/s [5] while the *D* of a protein of similar molecular weight (111 kilo Daltons) was 5.5 *μ*m^2^/s in the cytoplasm of *E. coli* [7]. From the small values of *D*s of large molecules in cellular compartments one may speculate that slow diffusion rates in heterogeneous catalytic reactions in living systems may generate characteristics that are typically associated with living systems. However, the significance of slow diffusion rates to living systems has not been demonstrated with an actual biological process.

It has been reported that microtubule assembly occurs in a stepwise fashion both *in vivo* [8, 9] and *in vitro* [10]. Kerssemakers et al. [10] hypothesized that the stepwise microtubule assembly resulted from a simultaneous addition of more than one tubulin dimer to the microtubule plus end in one assembly step but they did not address the nature of the pausing period between two successive steps. The periods in individual microtubule assembly steps and the pausing periods in [10] in general were less than one second and several seconds, respectively. Scheck et al. [11] conducted a study similar to that in [10] and found that microtubule growth occurred in steps smaller than those in [10]. Scheck et al. thus concluded that microtubule assembly occurs one tubulin dimer at a time. However, data in [11] still show that during certain periods of several-second long, microtubule growth still occurred in successive peaks with pausing periods between the successive peaks, even though the pausing periods were shorter than those in [10]. No explanation of the pausing phenomenon was given in [11]. Furthermore, the durations of the pausing periods in two different experimental conditions in [10] also appear to differ from each other. These quantitative differences may reflect a fundamental characteristic in heterogeneous catalysis that warrants further investigation.

In this study, it is shown that the differences in the duration of the rhythmic cycle (alternation of a fast growth period with a slow growth period in an absolutely positive microtubule assembly phase) of microtubule assembly described above can be explained by the differences in the critical concentration and the diffusion coefficient of tubulin molecules in the different experimental settings. These results support the hypothesis that slow diffusion rate of tubulin molecules relative to the assembly rate generates the phenomenon of alternating fast and slow growth periods in microtubule assembly. It is also suggested that such a mechanism generally operates in heterogeneous reactions *in vivo* to produce rhythmic reactions in biological systems.

## 2. Methods

The theoretical basis for this analysis is described in the following imaginary experiment in which an enzyme activity exists on a solid structure that catalyzes a heterogeneous reaction using a soluble protein substrate in the solution. If the reaction has occurred for a sufficient period of time, and the reaction rate is fast so that the diffusion rate of the soluble protein is a limiting factor for the reaction, a dynamic chemical concentration gradient of the soluble protein should be established with the lowest concentration at the reaction site and a slope of increasing concentration extending outward from the reaction site. It is predicted that the reaction will come to a temporary halt or a dramatic reduction when the soluble protein reaches or comes close to a critical concentration at the reaction site; the critical concentration, *C*_*c*_, is defined as such that below it the reaction ceases. Thus, this reaction system is expected to alternate between a fast reaction period *t*_*f*_ and a slow reaction period *t*_*s*_ (or even pause), and the sum of the averages of the two periods should be conceptually equal to the average diffusion time, *t*_*d*_, of the soluble protein along the concentration gradient. That is,

(1) $$\begin{array}{c} t_{d}=t_{f}+t_{s}. \end{array}$$

In this system, assuming one-dimensional diffusion to simplify the analysis, the diffusion time can be estimated by

(2) $$\begin{array}{c} t_{d}\approx \frac{x^{2}}{2D}, \end{array}$$

where *x* is the mean distance traveled by the diffusing protein molecules in one direction to the reaction site after elapsed time *t*_*d*_ and *D* is the diffusion coefficient of the protein in the solution. It is also noted here that *x* should be proportional to the length of the chemical gradient *L* when the system reaches *C*_*c*_ at the reaction site.

**Figure 1:**
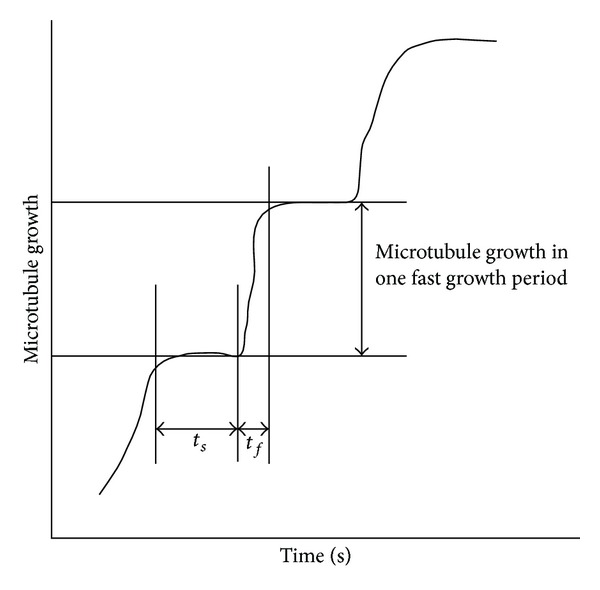
The durations of fast and slow growth periods of microtubule assembly and the length of microtubule growth in a fast growth period. The duration measurements were conducted with data in [10, 11], while the length measurements were only conducted with data in [11] since information on such lengths is available in [10].

**Figure 2:**
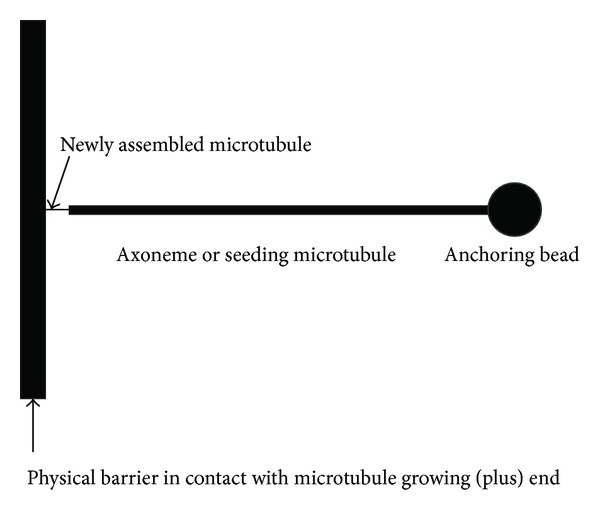
Schematic representation of the experimental setup for microtubule assembly. The anchoring bead was trapped in optical tweezers. Refer to [10, 11] for more details about the experimental apparatuses and reaction conditions.

To conduct the analysis, the average *t*_*f*_s and average *t*_*s*_s (Table 1) were extrapolated from the left halves of Figures 2(a) and 2(b) in [10] and the time periods of 2.8 seconds to 4.6 seconds, 6.8 seconds to 8.5 seconds, and 15 to 17.5 seconds for the experiment with GTP-tubulin in Figure 3(a) in [11]. These segments of the microtubule assembly curves were chosen because they consist of at least two peaks during one microtubule growth period so that one or more plateaus (corresponding to *t*_*s*_s) exist. A *t*_*f*_ period is from an early rising point of a peak to an early point at the peak, and a *t*_*s*_ is from an early point at the peak to an early rising point of the following peak, as illustrated in Figure 1. Two sets of experiments were conducted in [10]: one without the microtubule-associated protein XMAP215 and the other with XMAP215.

## 3. Results

All three average *t*_*f*_s in Table 1 are not statistically different from each other (*t*-test, *P* > 0.14) but the *t*_*s*_s in [10] are longer than that in [11] (*t*-test, *P* < 6 × 10^−4^). Furthermore, in [10], the *t*_*s*_ without XMAP215 is also longer than the *t*_*s*_ with XMAP215 (*t*-test, *P* = 0.03). These results indicate that the assembly reactions in all conditions occurred at a similar rate, and the variation in total diffusion time primarily derives from the variation in the duration of the slow growth period.

The average microtubule growth lengths per fast growth period in [10] were estimated, based on Figure 3(e) in the publication, to be 24 nm and 48 nm when without and with XMAP215, respectively. The average microtubule growth length per fast growth period in a particular condition is not given in [11]. The lengths of microtubule growth in the fast growth periods in Figure 3(a) in [11] were thus measured from the baseline of a preceding slow growth period to the topline of the subsequent fast growth period (Figure 1), and the average growth length per fast growth period is calculated to be 25.4 ± 3.7 nm (mean ± standard error, *n* = 15).

Considering that a microtubule consists of 13 protofilaments and each *αβ* tubulin dimer adds approximately 8 nm to the length of a protofilament, the growth of 24 nm and 48 nm of a microtubule consumes 39 ((24/8) × 13) and 78 ((48/8) × 13) tubulin dimers, respectively. Assuming that a uniform tubulin concentration gradient is established at the end of a fast growth period and microtubule assembly comes to a temporary halt due to reaching the critical tubulin concentration around the microtubule assembly point [12], the flux of tubulin during the fast growth period can be expressed as follows according to Fick's First Law:

(3) $$\begin{array}{c} J=-D\left(\frac{\partial C}{\partial X}\right)=-D\left[\frac{\left(C_{0}-C_{c}\right)}{L}\right], \end{array}$$

where *D* is the diffusion coefficient, *C*_0_ is the tubulin concentration outside the tubulin gradient, and *L* is the length of the tubulin concentration gradient. The flux can also be calculated by dividing the amount of tubulin consumed in a fast growth period by the area of the microtubule assembly site and the duration of the fast growth period. Then (3) is turned into

(4) J−XMAP215=−D[(C0−Cc−XMAP215)L−XMAP215]=[39/(6.022×1023)](atf−XMAP215),

(5) J+XMAP215=−D[(C0−Cc+XMAP215)L+XMAP215]=[78/(6.022×1023)](atf+XMAP215),

where −XMAP215 and +XMAP215 denote the two experiments in [10] respectively, and *a* is the area parameter around the microtubule assembly point and is intrinsic to the geometric form of the microtubule assembly point. 6.022 × 10^23^ is the Avogadro's Constant.

The microtubule assembly conditions without and with XMAP215 are assumed to be comparable to the previously described conditions for microtubule assembly without and with microtubule-associated proteins, respectively. *C*_*c*−XMAP215_ and *C*_*c*+XMAP215_ are thus estimated to be 4 *μ*M [12] and 2 *μ*M [13, 14], respectively. Using the *t*_*f*_ values in Table 1, letting the average *C*_0_ = (5 + 20)/2 = 12.5 *μ*M [10, online supplementary information], and dividing (4) by (5), the following is obtained

(6) $$\begin{array}{c} \frac{\left[\left(12.5-4\right)/L_{-\text{XMAP}215}\right]}{\left[\left(12.5-2\right)/L_{+\text{XMAP}215}\right]}=\frac{\left(39\times 0.63\right)}{\left(78\times 0.55\right)}. \end{array}$$

Consolidating (6), then

(7) $$\begin{array}{c} \frac{L_{+\text{XMAP}215}}{L_{-\text{XMAP}215}}=0.71,\;\text{or}\;\frac{{L_{+\text{xmap}215}}^{2}}{{L_{-\text{xmap}215}}^{2}}=0.5. \end{array}$$

It is presumed that the average diffusion distance *x* is proportional to the tubulin concentration gradient length *L*; then

(8) $$\begin{array}{c} \frac{{x_{+\text{XMAP}215}}^{2}}{{x_{-\text{XMAP}215}}^{2}}=\frac{{L_{+\text{XMAP}215}}^{2}}{{L_{-\text{XMAP}215}}^{2}}=0.5. \end{array}$$

From (2) and (8), the following is obtained

(9) $$\begin{array}{c} \frac{t_{d+\text{XMAP}215}}{t_{d-\text{XMAP}215}}=\frac{{x_{+\text{XMAP}215}}^{2}}{{x_{-\text{XMAP}215}}^{2}}=0.5. \end{array}$$

The experimental result from *t*_*d*_ values in Table 1 is

(10) $$\begin{array}{c} \frac{t_{d+\text{XMAP}215}}{t_{d-\text{XMAP}215}}=\frac{2.96}{4.4}=0.67. \end{array}$$

The calculated value of *t*_*d*+XMAP215_/*t*_*d*−XMAP215_ is quite close to that of the experimental *t*_*d*+XMAP215_/*t*_*d*−XMAP215_, supporting the assumption that the periodic slowdowns in microtubule assembly are caused by the periodic occurrences of insufficient flux of tubulin that arise from the slow diffusion rate relative to the reaction rate.

In the microtubule assembly process described in [11], isolated microtubule fragments were used to seed the microtubule growth. These seeding microtubule fragments should have retained a high level of microtubule-associated proteins. It is thus assumed that *C*_*c*_ ≈ 2 *μ*M in [11]. Because *C*_0_ = 5 *μ*M in [11], the tubulin flux at the microtubule assembly site during the fast growth period is

(11) $$\begin{array}{c} J_{\text{Schek}}=-\frac{D_{\text{Schek}}\left(5-2\right)}{L_{\text{Schek}}}=\frac{\left[39/\left(6.022\times 10^{23}\right)\right]}{\left(at_{f\text{Schek}}\right)}. \end{array}$$

Dividing (4) by (11), the following is obtained

(12) J−XMAP215JSchek=(8.5D−XMAP215/L−XMAP215)(3DSchek/LSchek)=tfSchektf−XMAP215.

Using the *t*_*f*_ values in Table 1 to consolidate (12), then

(13) $$\begin{array}{c} \frac{L_{\text{Schek}}}{L_{-\text{XMAP}215}}\approx \frac{0.28D_{\text{Schek}}}{D_{-\text{XMAP}215}} \end{array}$$

or

(14) $$\begin{array}{c} \frac{{L_{\text{Schek}}}^{2}}{{L_{-\text{XMAP}215}}^{2}}\approx \frac{{0.078D_{\text{Schek}}}^{2}}{{D_{-\text{XMAP}215}}^{2}}. \end{array}$$

From (2) and based on *L*_Schek_^2^/*L*_−XMAP215_^2^ = *x*_Schek_^2^/*x*_−XMAP215_^2^, the following is obtained

(15) $$\begin{array}{c} \frac{t_{d\text{Schek}}}{t_{d-\text{XMAP}215}}=\frac{\left({L_{\text{Schek}}}^{2}D_{-\text{XMAP}215}\right)}{\left({L_{-\text{XMAP}215}}^{2}D_{\text{Schek}}\right)}. \end{array}$$

From (14) and (15), the following is derived

(16) $$\begin{array}{c} \frac{t_{d\text{Schek}}}{t_{d-\text{XMAP}215}}\approx \frac{0.078D_{\text{Schek}}}{D_{-\text{XMAP}215}}. \end{array}$$

For one molecular species in different solutions at the same temperature, its *D* values are in an inverse relationship with the viscosity of the solutions. The solution used in [10] for microtubule assembly is described as “very viscous”; the solution contained up to four times of tubulin concentration of that in [11]. In addition, the assembly solution in [10] contained 0.5–1% bovine serum albumin that was absent in the assembly solution in [11]. Therefore, the viscosity of the assembly solution in [10] is expected to be significantly higher than that in [11]; that is, *D*_Schek_ > *D*_−XMAP215_. Based on the values in Table 1, the experimental *t*_*d*Schek_/*t*_*d*−XMAP215_ becomes

(17) $$\begin{array}{c} \frac{t_{d\text{Schek}}}{t_{d-\text{XMAP}215}}=\frac{0.98}{4.4}=0.22. \end{array}$$

Let *t*_*d*Schek_/*t*_*d*−XMAP215_ in (16) and (17) be the same; then

(18) $$\begin{array}{c} \frac{0.078D_{\text{Schek}}}{D_{-\text{XMAP}215}}=0.22 \end{array}$$

or

(19) $$\begin{array}{c} D_{\text{Schek}}\approx 2.8D_{-\text{XMAP}215}. \end{array}$$

Equation (19) gives a plausible value of *D*_Schek_ in relation to *D*_−XMAP215_, considering the viscosity differences in the two microtubule assembly solutions.

The above analysis is based on the diffusion flux in one dimension. To scale it up to a three-dimensional flux, it is assumed that there are a total of *n* paths for tubulin dimers to travel towards the microtubule assembly site. It is predicted that these paths form the 3D configuration of the tubulin flux that has a geometric focus at the microtubule assembly site and is radially symmetrical around the growing microtubule. If the tubulin concentration gradient lengths of these paths are *L*_1_, *L*_2_,…, *L*_*n*_, there should exist a certain quantitative relationship between *L*_1_ and other *L*s; that is, *L*_2_ = *b*_2_ *L*_1_, *L*_3_ = *b*_3_ *L*_1_,…, *L*_*n*_ = *b*_*n*_ *L*_1_. The values of the *b*s are determined by the shape of the 3D configuration of the tubulin flux. Therefore,

(20) The  average  L3D=(L1+L2+L3+⋯+Ln)n=L1(1+b2+b3+⋯+bn)n.

Because the experimental apparatus in [10] is essentially the same as that in [11] (Figure 2), the 3D configurations of the tubulin fluxes in the three reaction conditions discussed in this report should be of the same shape but different sizes; that is, they have the same *b* values with respect to the same paths. The earlier discussed *L*_−XMAP215_, *L*_+XMAP215_, and *L*_Schek_ can be considered the tubulin concentration gradient lengths of the same path in the three tubulin 3D fluxes, respectively. The average *L*s of the three 3D tubulin fluxes can then be expressed as

(21) Average  L−XMAP215-3D  =L−XMAP215(1+b2+b3+⋯+bn)n,Average  L+XMAP215-3D  =L+XMAP215(1+b2+b3+⋯+bn)n,Average  LSchek-3D  =LSchek(1+b2+b3+⋯+bn)n.

Equation (21) indicates that the ratios among *L*_−XMAP215_, *L*_+XMAP215_, and *L*_Schek_ equal the ratios among the average *L*_−XMAP215-3D_, *L*_+XMAP215-3D_, and *L*_Schek-3D_. The results obtained from (9) and (16), therefore, also apply to the 3D tubulin flux situation.

A further test of the proposed diffusion mechanism that underlies the microtubule assembly rhythmic behavior is that, during the *t*_*f*_ period, whether the consumption of tubulin dimers by the microtubule assembly process can temporarily reduce the tubulin concentration around the assembly site to the level of tubulin critical concentration in the three specific conditions discussed in this report. If so, it is taken as the indication that the uniform tubulin gradient near the assembly site was temporarily disrupted during *t*_*f*_ period and it needs to be reestablished during the *t*_*s*_ period before the next round of microtubule assembly. In other words, repetitions of a temporary disruption of the tubulin gradient followed by reestablishment of the gradient manifest into a rhythmic microtubule assembly behavior. To test the possibility of such temporary disruption in the tubulin gradient, a hemisphere with a radius *R* of the length of the average diffusion distance during the time of *t*_*f*_ and the assembly site as the center is deemed a relevant space in which the disruption takes place. A hemisphere is considered here because the barrier in the experimental apparatuses should block the tubulin diffusion from the left side of the barrier (Figure 2). If the tubulin concentration within the hemisphere is reduced to the tubulin critical concentration, the number of consumed tubulin dimers, *N*, is

(22) N=(23)πR3[(Cedge−Ccenter)2](6.022×1023) ≈(23)πR3[(R/x)(C0−Cc)2](6.022×1023),

where *C*_edge_ and *C*_center_ are the tubulin concentrations at the center and edge of the hemisphere before the disruption of the tubulin gradient, respectively, and *x* is the average diffusion distance during the time of *t*_*d*_.

From (2), *R* = (2*t*_*f*_ *D*)^1/2^, and *x* = (2*t*_*d*_ *D*)^1/2^, (22) then becomes

(23) $$\begin{array}{c} N\approx 2.96\left(6.022\times 10^{23}\right){t_{f}}^{2}{t_{d}}^{-1/2}D^{3/2}\left(C_{0}-C_{c}\right). \end{array}$$

When *D* = 0.07 *μ*m^2^/s in [10] and *D* = 0.07 × 2.8 *μ*m^2^/s in [11] (see (19)), using the *t*_*f*_ and *t*_*d*_ values in Table 1 and previous *C*_0_ and *C*_*c*_ values, (23) is solved for the three experimental conditions to arrive at, respectively,

(24) $$\begin{array}{c} N_{-\text{XMAP}215}\approx 40,\\ N_{+\text{XMAP}215}\approx 80,\\ N_{\text{Schek}}=91. \end{array}$$

The *N*_−XMAP215_ and *N*_+XMAP215_ values are very close to the flux values of 39 and 78 that were estimated based on the average lengths of microtubule growth per fast growth period in the two conditions, respectively. *N*_Schek_ is approximately two times the estimated flux value of 39 in the corresponding condition. The *R* values in the three cases are 0.28 *μ*m, 0.30 *μ*m, and 0.42 *μ*m, respectively, and the corresponding *x* values are 0.78 *μ*m, 0.63 *μ*m, and 0.62 *μ*m. These values seem to be within reasonable ranges for the experimental conditions, which suggest that, within the *t*_*f*_ period, the microtubule assembly process is capable of depleting the tubulin dimers to a concentration at or near the critical concentration of microtubule assembly. The depicted *D* values that produce the desired *N* values are smaller than the *D* value of tubulin dimers in the cytoplasm in sea urchin [5], but they may be close to the actual *D* values in the *in vitro* experimental conditions. The *N*_Schek_ value is somewhat higher than the desired result, but it can be potentially attributed to imprecise estimates of the other parameters in (23). In conclusion, the calculations demonstrate that it is at least plausible that the slow diffusion rates relative to the microtubule assembly rates can generate the pattern of alternating fast and slow growth periods of microtubule assembly.

## 4. Discussion

In biological systems, the diffusion coefficients of large molecules are small due to the large sizes and variable shapes of the molecules, the high viscosity of the fluid, and other more complex molecular interactions. It is not difficult to envision that the proposed mechanism based on the microtubule assembly process can be generally applicable to heterogeneous reactions in biological systems. Many molecular processes in biological systems are known to be rhythmic. For example, levels of secondary messenger molecules oscillate in cells [15], ion channels generate rhythmic electrical activities in neurons and cardiac cells [16], and single immobilized enzyme molecule exhibits rhythmic catalysis [17]. Whether and how slow diffusion rates relative to reaction rates play a role in generating these rhythmic behaviors remain to be investigated. It will be also very interesting to explore whether and how multiple rhythmic reactions at the individual reaction sites integrate into rhythmic behaviors and predictable patterns at a higher (e.g., system) level.

In the studies of the catalytic kinetics of immobilized single enzyme molecules, it was revealed that the catalytic reactions occurred in periods of high activity alternating with periods of low activity and the “waiting times” (in reference to the durations of the low activity periods by the investigators) fall into a broad time scale of milliseconds to seconds [17, 18]. It has been hypothesized that an enzyme molecule should have thousands of conformational states so that the waiting time can vary from milliseconds to seconds [17, 18]. It is argued here that the rhythmic nature of single enzyme catalysis with a broad range of waiting time can be simply explained by the same mechanism proposed for microtubule assembly in this study. The enzyme-substrate complex can be safely assumed to be a dynamic union, which undergoes separation-and-reunion cycles mainly due to the thermal dynamic movement of the substrate molecule since the enzyme molecules in the studies were immobilized. This dynamic process is expected to have a broad time scale that depends on the variable distance of the separated substrate molecule to the enzyme molecule; the waiting time range should be in proportion to the square of the distance range. Also importantly, the diffusion mechanism predicts that the waiting time should be reduced along with the increase of the substrate concentration, whereas it is difficult to fathom that the number of the conformational states should be reduced by the increase of the substrate concentration. Indeed, English et al. [17] found that the waiting time was decreased along with the increase of the substrate concentration.

Because nothing is known about the critical tubulin concentration for microtubule disassembly, this study is limited to the microtubule assembly process. However, microtubule disassembly also appears to alternate between fast and slow periods and overall occurs faster than microtubule assembly [10, 11]. Therefore, the degradation of a biological structure may also follow a similar mechanism as proposed for the synthesis of a biological structure, although the kinetics may be different from that of synthesis.

The length of *in vitro* microtubule growth in one fast growth period is much less than one unit (approximately 350 nm) of microtubule elongation observed in the *in vivo* spindle elongation process [8, 9]. It suggests that either the *in vivo* fast growth period of microtubule assembly is much longer than the *in vitro* fast growth period or there is another level of pause in assembly after a fixed number of fast and slow growth periods similar in duration to those observed *in vitro*. To determine which of the two scenarios occurs *in vivo*, it requires studies of microtubule assembly in cells at sufficient time and length resolutions.

## 5. Conclusion

This study of two *in vitro* microtubule assembly cases demonstrates that small diffusion coefficients of a reactant can lead to rhythmic behavior of the reaction in a heterogeneous reaction system.

## Figures and Tables

**Table 1:** Average durations (in second) of fast growth periods (*t*_*f*_) and slow growth periods (*t*_*s*_) in *in vitro* microtubule assembly and tubulin diffusion times (*t*_*d*_ = *t*_*f*_ + *t*_*s*_).

Mean *t*_*f*_ ± standard error	Mean *t*_*s*_ ± standard error	Mean *t*_*d*_	Seed for microtubule assembly	Reference
0.55 ± 0.09 (*n* = 5)	3.85 ± 0.57 (*n* = 17)	4.4	Axoneme − XMAP215	[10]
0.63 ± 0.11 (*n* = 6)	2.33 ± 0.37 (*n* = 11)	2.96	Axoneme + XMAP215	[10]
0.44 ± 0.04 (*n* = 8)	0.54 ± 0.08 (*n* = 6)	0.98	Microtubule fragments	[11]

*n*: number of samples measured.

## References

[1] Stenberg M, Stiblert L, Nygren H (1986). External diffusion in solid-phase immunoassays. *Journal of Theoretical Biology*.

[2] van Roon JL, Arntz MM, Kallenberg AI (2006). A multicomponent reaction-diffusion model of a heterogeneously distributed immobilized enzyme. *Applied Microbiology and Biotechnology*.

[3] Young ME, Carroad PA, Bell RL (1980). Estimation of diffusion coefficients of proteins. *Biotechnology and Bioengineering*.

[4] Mastro AM, Babich MA, Taylor WD, Keith AD (1984). Diffusion of a small molecule in the cytoplasm of mammalian cells. *Proceedings of the National Academy of Sciences of the United States of America*.

[5] Salmon ED, Saxton WM, Leslie RJ (1984). Diffusion coefficient of fluorescein-labeled tubulin in the cytoplasm of embryonic cells of a sea urchin: video image analysis of fluorescence redistribution after photobleaching. *Journal of Cell Biology*.

[6] Verkman AS (2002). Solute and macromolecule diffusion in cellular aqueous compartments. *Trends in Biochemical Sciences*.

[7] Nenninger A, Mastroianni G, Mullineaux CW (2010). Size dependence of protein diffusion in the cytoplasm of *Escherichia coli*. *Journal of Bacteriology*.

[8] Yang M, Ma H (2001). Male meiotic spindle lengths in normal and mutant Arabidopsis cells. *Plant Physiology*.

[9] Yang M, Wang Y (2011). A model for discrete spindle elongation. *Cell Cycle*.

[10] Kerssemakers JW, Munteanu EL, Laan L, Noetzel TL, Janson ME, Dogterom M (2006). Assembly dynamics of microtubules at molecular resolution. *Nature*.

[11] Schek HT, Gardner MK, Cheng J, Odde DJ, Hunt AJ (2007). Microtubule assembly dynamics at the nanoscale. *Current Biology*.

[12] Mitchinson T, Kirschner M (1984). Dynamic instability of microtubule growth. *Nature*.

[13] Olmsted JB, Marcum JM, Johnson KA, Allen C, Borisy GG (1974). Microtuble assembly: some possible regulatory mechanisms. *Journal of Supramolecular Structure*.

[14] Gaskin F, Cantor CR, Shelanski ML (1974). Turbidimetric studies of the in vitro assembly and disassembly of porcine neurotubules. *Journal of Molecular Biology*.

[15] Willoughby D, Cooper DMF (2006). Ca^2+^ stimulation of adenylyl cyclase generates dynamic oscillations in cyclic AMP. *Journal of Cell Science*.

[16] Thon S, Schmauder R, Benndorf K (2013). Elementary functional properties of single HCN2 channels. *Biophysical Journal*.

[17] English BP, Min W, van Oijen AM (2006). Ever-fluctuating single enzyme molecules: Michaelis-Menten equation revisited. *Nature Chemical Biology*.

[18] Yang H, Luo G, Karnchanaphanurach P (2003). Protein conformational dynamics probed by single-molecule electron transfer. *Science*.

